# Long-term effectiveness of the diabetes conversation map program: A prepost education intervention study among type 2 diabetic patients in Taiwan: Erratum

**DOI:** 10.1097/MD.0000000000010673

**Published:** 2018-05-04

In the article, “Long-term effectiveness of the Diabetes Conversation Map Program: A prepost education intervention study among type 2 diabetic patients in Taiwan”,^[1]^ which appeared in Volume 96, Issue 36 of *Medicine*, affiliation b should appear as:

^b^Department of Metabolism and Endocrinology, Dalin Tzu Chi Hospital, Buddhist Tzu Chi Medical Foundation, Dalin, Chiayi, Taiwan and School of Medicine, Tzu Chi University, Hualien City, Taiwan.

## References

[1] Hung JY Chen PF Livneh H Long-term effectiveness of the Diabetes Conversation Map Program: A prepost education intervention study among type 2 diabetic patients in Taiwan. *Medicine.* 2017 96:e7912. 28885345 10.1097/MD.0000000000007912 PMC6392651

